# Proper doses of brassinolide enhance somatic embryogenesis in different competent Korean pine cell lines during embryogenic callus differentiation

**DOI:** 10.3389/fpls.2024.1330103

**Published:** 2024-01-23

**Authors:** Shuai Nie, Yong Yan, Yue Wang, Shanshan Liu, Wenhui Guo, Ling Yang, Hailong Shen

**Affiliations:** ^1^ College of Forestry, Northeast Forestry University, Harbin, China; ^2^ State Key Laboratory of Tree Genetics and Breeding (Northeast Forestry University), Harbin, China; ^3^ State Forestry and Grassland Administration Engineering Technology Research Center of Korean Pine, Harbin, China

**Keywords:** *Pinus koraiensis*, brassinolides, antioxidant enzymes, hormones, embryogenic callus, somatic embryogenesis

## Abstract

Somatic embryogenesis of Korean pine (*Pinus koraiensis* Sieb. Et Zucc.), an ecologically and econimically very important conifer species, was hindered by the gradually weakens and fast runaway of the embryogenicity and embryo competence of the embryogenic callus. Brassinolide (BL) has shown the enhancing capability of somatic embryo regeneration. For checking the function of BL in this issue, we applied different concentrations of BL to Korean pine callus materials exhibiting different embryogenic capacities and subsequently monitored the physiological alterations and hormone dynamics of the embryogenic callus. Our study revealed that calli with different embryogenic strengths responded differently to different concentrations of BL, but the effect after the addition of BL was very uniform. The addition of BL during the proliferation phase of embryogenic callus may help to stimulate the biological activity of callus during the proliferation process and improve the level of cell metabolism, which is accompanied by a reduction in storage substances. BL could reduce the level of endogenous auxin IAA in embryogenic callus and increase the level of abscisic acid to regulate cell division and differentiation. In addition, the MDA content in the callus was significantly decreased and the activity of antioxidant enzymes was significantly increased after the addition of BL. During the proliferation of embryogenic callus, BL was added to participate in the metabolism of phenylpropane in the cells and to increase the activity of phenylalanine ammonia-lyase and the content of lignin in the cells. We deduced that the proper doses of BL for Korean pine embryogenic callus culture were as follow: calli with low, high and decreasing embryogenicity were subcultured after the addition of 0.75 mg/L, 0.35 mg/L, 2.00 mg/L BL, respectively, during proliferation culture stage.

## 1 Introduction

Brassinolide (BL) is the first discovered form of brassinosteroids (BRs) with the most bioactive properties and BRs play a crucial role in various aspects of plant growth and development, including cell growth and division, vascular bundle differentiation, cell redifferentiation, and growth under stress conditions (Kim et al., 2009; Wei and Li, 2016; Bajguz et al., 2020; Kour et al., 2021; Singh et al., 2021; Manghwar et al., 2022). The regeneration and multiplication rate of *in vitro* cultured material could be improved by the addition of BRs to the medium for callus induction, somatic embryogenesis and embryogenic callus formation (Singh et al., 2021); 4 types of hybrid sweetgum (*Liquidambar styraciflua* × *L. formosana*) with different embryogenic ties should be highly correlated with endogenous BRs; and BR concentrations of the samples should be positively correlated with the expression of genes associated with somatic embryos (Zhao et al., 2022). In general, the general aspects of BRs have been studied in relative detail (Singh et al., 2021; Chen et al., 2022), but Somatic embryogenesis (SE) involves the use of BRs, including their application effects and physiological and genetic mechanisms (Kim and Moon, 2014; Chone et al., 2018; Zhao et al., 2022). Research has shown that BRs can regulate cell elongation by altering cell walls. They can also influence cell wall formation by regulating the genes responsible for cell wall synthesis (Xie et al., 2011). The results of the study indicate that the application of BRs to cells results in an increase in the expression of cyclin CycD3, which in turn influences the process of cell division (Hu et al., 2000). The formation of embryogenic cells in Korean pine is primarily the result of asymmetric cell division (Peng et al., 2022a).Therefore, the increase in cell volume during the process of cell proliferation and division may indirectly increase the probability of asymmetric division.

Coniferous tree species are widespread throughout the world and occupy a dominant position in large forest ecosystems. They play a crucial role in maintaining biodiversity and storing carbon. Among these species, Korean pine (*Pinus koraiensis* Sieb. et Zucc.) is particularly noteworthy due to its high economic and ecological value (Zhao et al., 2014). Korean pine forests are widely distributed in northeast China, the Russian Far East, the Korean Peninsula, and central Japan (Jin et al., 2017). In China, Korean pine has been classified as an endangered species due to excessive deforestation. However, the selection and breeding of improved Korean pine cultivars is limited, and it is difficult to propagate these cultivars, resulting in a shortage of improved Korean pine material. Somatic embryogenesis, a system for plant propagation and gene transformation (Méndez-Hernández et al., 2019), has the potential for large-scale propagation and has already been successfully used in the propagation of many tree species (Lelu-Walter et al., 2013; Heringer et al., 2018; Egertsdotter et al., 2019), making it a promising solution to address the shortage of improved Korean pine cultivars. The efficiency of SE in conifers is hampered by a gradual decline in its ability with increasing duration of the subculture cycle, as observed in most conifers (Trontin et al., 2011). This poses a challenge in the application of SE for conifer breeding and for the rapid and large-scale propagation of improved material.

The callus of Korean pine has been successfully obtained from both mature and immature zygotic embryos (Bozhkov et al., 1997; Gao et al., 2017). In addition, plant regeneration has been successfully achieved from immature zygotic embryos (Gao et al., 2020). Despite its potential benefits, the application of Korean pine SE in actual production is limited by unfavorable aspects. One of the major main problems in large-scale production of Korean pine SE is hindered by problems associated with the following issues (Gao et al., 2022): the possibility of embryogenic callus losing its potential for SE after undergoing extended subculture, which has already been mentioned by Trontin et al. (2011). To counteract this, one must intervene with exogenous hormones to reduce the embryogenic loss of embryogenic callus or improve the stress resistance of embryogenic callus to maintain the number of embryogenic callus (Ren et al., 2022). This approach offers a solution to the problem of pine SE, as the specific steps by which this problem occurs have been identified.

At the somatic embryogenesis stage, a higher ratio of IAA to ABA is beneficial for the formation of somatic embryos (Peng et al., 2022a). Furthermore, this ratio can serve as a reliable indicator of the capacity for embryogenesis in explants (Grzyb et al., 2017). In our previous research, we discovered a correlation between the concentrations of IAA and ABA in callus with different embryogenic capabilities (Peng et al., 2022b). In plant cells, BL triggers the expression of the BRU1 gene, which leads to a loosening of the cell wall and promotes cell elongation (Zurek and Clouse, 1994). This biological activity is stronger than that of auxin hormones, such as 2,4-D, IAA, NAA, and other similar hormones, and these hormones act synergistically with each other (Mandava, 1988; Zurek and Clouse, 1994). The mechanism of BL induced by growth is not dependent on auxin; BR acts on the inner tissue during elongation, and alters the physical properties of the cell wall (Suleman, 1994). However, BL cannot replace other plant growth regulators. BL promotes cell elongation by acting on the inner tissue and changing the physical properties of the cell wall (Xiong et al., 2021). This effect is specific to BL and has been observed in various studies (Rumi et al., 1994). BL plays a crucial role in both the synthesis of the cell wall and the regulation of plant cell elongation (Rao and Dixon, 2017), and the associated regulatory mechanism is closely linked to the phenylpropane metabolic pathway in plant cells.

In this study, three types of embryogenic calli with known embryogenic vigor from Korean pine trees were used as experimental material. Different concentrations of BL were added to the calli during callus proliferation to investigate the effects of BL on the content of storage substances, antioxidant enzyme activity, the content of key hormones for somatic embryo induction, and the key enzymes and products of a phenylpropane metabolic pathway in embryogenic callus at different treatment times. This study sheds light on the mechanism of BL to maintain a high level of embryogenic ability during the callus proliferation phase and provides a solution to a crucial aspect of Korean pine somatic embryogenesis. Our research provides a basis for the use of sterol hormones to enhance the ability to transform somatic embryos from callus while maintaining their embryogenicity. This could serve as a reference for SE in other conifer species.

## 2 Materials and methods

### 2.1 Plant materials

Immature cones were collected from mature Korean pine trees (*Pinus koraiensis*) in Lushuihe Seed Orchard of Jilin Province, China, in July 2019. Embryogenic calli were induced from zygotic embryo explants and classified according to strength and weakness. The callus was then stored in liquid nitrogen using cryopreservation technology. Three cell lines were selected for this experiment based on their known embryogenic ability. The embryogenic strength of the materials was classified based on the average number of somatic embryos produced per gram of fresh weight (FW) of embryogenic callus. A genotype with weak embryogenic ability (genotype A) was defined as an embryogenic callus that produced approximately 100 somatic embryos per gram FW of embryogenic callus. On the other hand, a genotype with strong embryogenic ability (genotype B) was defined as an embryogenic callus that produced more than 200 somatic embryos per gram of FW embryogenic callus. In addition, genotype C refers to a material with an initially strong embryogenic capacity that declines after long-term passage (more than 6 months). In general, the number of somatic embryos produced per gram of fresh weight does not exceed 50 per gram of FW embryogenic callus.

### 2.2 Preparation of proliferation culture medium

The proliferation medium used in this experiment was mLV basal medium, supplemented with 2 mg/L 2,4-D, 0.5 mg/L 6-BA, 25 g/L sucrose, 500 mg/L acid hydrolyzed casein, and 500 mg/L L-glutamine. The curing agent used was 4 g/L gellan gum, and the pH of the medium was adjusted to 5.8 before autoclaving. In the experimental group, a brassinolide (BL) solution was added to the sterilized medium, which was previously prepared, using a sterile disposable syringe and a Filter sterilizer (Millex - GP Syringe Filter Unit, 0.22-μm, Merck KGaA, Darmstadt, Germany). The concentrations of the BL solution used were 0.05, 0.25, 0.35, 0.50, 0.75, 1.00, 1.25, 1.50, and 2.00 mg/L, while the medium without BL served as a control group. After inoculation, the dark cultivation method was used, and the cultivation temperature was 25°C.

### 2.3 Culture of embryogenic calli

After 7 or 14 days of proliferation and culture, the embryogenic callus was packed and a sample was taken for the test materials. Three replicates were made for each genotype and treatment, and 0.5 g of fresh callus was collected for each replicate. The collected embryogenic callus samples were stored in 1.8 mL cryogenic tubes, labeled, and stored in a refrigerator at -80°C for later use.

### 2.4 Conversion of somatic embryos from embryogenic calli after brassinolide addition

The embryogenic calli stored in liquid nitrogen were evenly distributed in culture dishes for the experimental and control groups. After 7 and 14 days of proliferation and culture, the calli developed into somatic embryos using mLV (Coolaber, Beijing, China) basal medium supplemented with 21 mg/L ABA, 1.0 g/L activated carbon, 68 g/L sucrose, 500 mg/L acid hydrolyzed casein, and 500 mg/L L-glutamine. The medium was hardened with using 12 g/L gellan gum, and had with a pH of 5.8 before autoclaving.

To prepare the embryogenic calli, they were suspended in mLV medium containing 25 g/L sucrose. Subsequently, 3 mL of the suspension cultures containing 70 mg embryogenic callus were transferred to a sterile filter paper disc in a Buchner funnel. The liquid medium was extracted using a filter bottle and vacuum pump, resulting in an even distribution of the embryogenic callus on the filter paper disc. The filter paper discs containing the embryogenic calli were then transferred to a maturation medium and cultured for eight weeks in the dark at 25°C without subculture. Each treatment was repeated five times. The dark culture method with a cultivation temperature of 25°C was used for inoculation.

### 2.5 Biochemical analysis of embryogenic calli cultured on media with different concentrations of brassinolide

Physiological tests were performed on embryogenic callus after brassinolide treatment. Determination methods for soluble protein, soluble sugar, starch, POD, SOD, and CAT were performed according to the procedure described by Peng et al. (2020). The MDA content was determined using the MDA content kit purchased from Suzhou Keming Biological Co., Ltd. for the micro methods. The endogenous hormones were measured in the embryogenic callus treated with brassinolide. The frozen tube containing the sample was removed, stored on dry ice, and sent to Shanghai Enzyme Biotechnology Co., Ltd. for determination. The ELISA method was used to determine the endogenous IAA, ABA, BL, phenylalanine ammonia-lyase (PAL), and woodiness of the sample. The determination of lignin was also performed.

### 2.6 Data statistics and analysis

Experimental data were compiled using Microsoft Excel 2019, and statistical analysis was performed using SPSS (v26.0, SPSS Inc., Cary, NC, USA) software. Duncan’s multiple comparisons (Duncan), one-way ANOVA (One-way ANOVA), and Pearson correlation analysis were conducted on the callus proliferation rate with a significant difference level of P = 0.05. Mapping was performed using Origin Pro 2021(9.8.0.200) software.

## 3 Results

### 3.1 Effect of brassinolide on somatic embryo maturation and transformation of embryogenic calli of Korean pine

**Figure 1:**
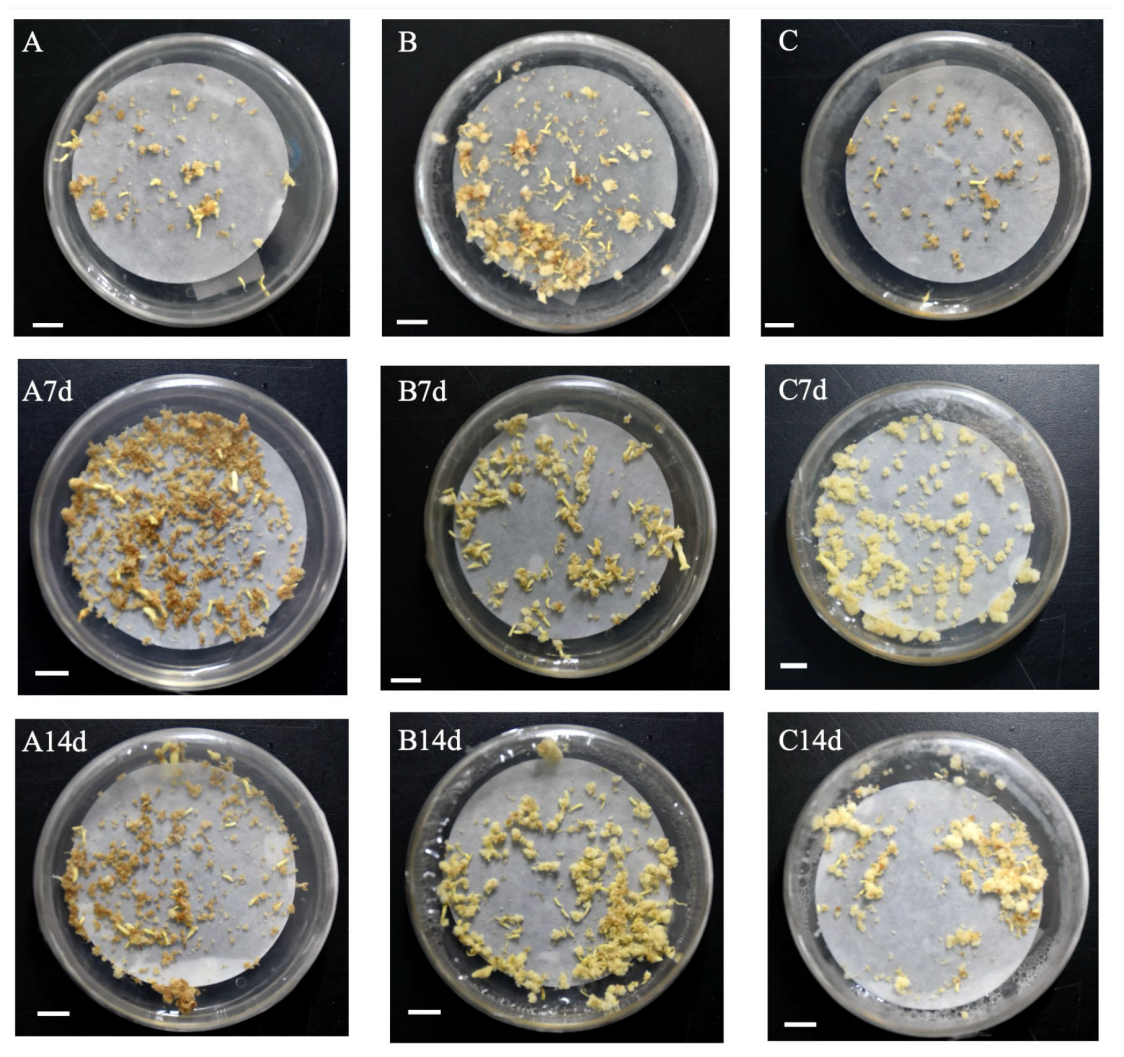
Documentation callus of Korean pine into somatic embryos under different BL treatments (bar=1 cm). **(A)** genotype A was not treated with BL for 14 days; **(B)** genotype B was not treated with BL for 14 days; **(C)** genotype C was not treated with BL for 14 days. A7d-genotype A was treated with 0.75 mg/L BL for 7 days; A14d-genotype A was treated with 0.75 mg/L BL for 14 days; B7d-genotype B was treated with 0.35 mg/L BL for 7 days; B14d-genotype B was treated with 0.35 mg/L BL was added for 14 days; C7d-genotype C was treated with 2.00 mg/L BL for 7 days; C14d-genotype C was treated with 2.00 mg/L BL for 14 days.

Somatic embryo transformation of embryogenic calli was carried out by adding brassinolide during the proliferation stage. In the control groups of genotypes A and B, the number of somatic embryo transfers of more than 14 days was observed after 7 days of culture. When comparing the number of somatic embryos at different concentrations for 7 days and 14 days, BL was found to have the best effect on genotype B, leading to a 226.57% increase in somatic embryo production when BL was added for 7 days (0.05 mg/L, 7 d) (**Table 1**). After 14 days of culture, the highest increase of 152.98% (0.35 mg/L, 14 days) was observed. In genotype A, the highest increase in somatic embryo production was 231.14% when BL was added (0.75 mg/L, 7 days), and the highest increase was 258.77% after 14 days of culture (0.75 mg/L, 14 days) (**Table 1**). When BL was added to genotype C, the highest yield of somatic embryos was observed by 90.00% after 7 days of culture, and the highest increase was 132.79% after 14 days of culture (2.00 mg/L, 14 days) (**Table 1**). We documented the growth of partial somatic embryos and the number of somatic embryo transitions for the three genotypes before and after adding BL. The representative sample of these findings is illustrated in **Figure 1**.

**Table 1:** Means (± SD) indicate the number of somatic embryo transfers of three genotypes of embryogenic calli at varying concentrations of brassinolide treatment (number·g^-1^ FW embryogenic callus).

Concentrations of Brassinolide/mg/L	Genotype A		Genotype B		Genotype C	
	7d	14d	7d	14d	7d	14d
0	167 ± 133b	114 ± 38c	286 ± 38b	167 ± 68bcd	10 ± 8a	62 ± 16a
0.05	43 ± 14bc	90 ± 8cd	648 ± 95a	176 ± 44bc	0 ± 0a	0 ± 0c
0.25	10 ± 8c	229 ± 38ab	95 ± 36def	229 ± 62ab	19 ± 8a	29 ± 14b
0.35	29 ± 14c	167 ± 87bc	214 ± 43bc	257 ± 43a	19 ± 22a	0 ± 0c
0.50	71 ± 25bc	148 ± 22bc	24 ± 22f	129 ± 25cd	14 ± 14a	0 ± 0c
0.75	386 ± 171a	295 ± 101a	162 ± 36cd	90 ± 44d	5 ± 8a	5 ± 8c
1.00	0 ± 0c	71 ± 62cd	152 ± 59cde	176 ± 44bc	19 ± 8a	5 ± 8c
1.25	0 ± 0c	0 ± 0d	71 ± 14ef	148 ± 44bcd	10 ± 8a	0 ± 0c
1.50	48 ± 8bc	138 ± 44bc	10 ± 8f	100 ± 29cd	5 ± 8a	10 ± 8bc
2.00	114 ± 52bc	138 ± 8bc	267 ± 64b	0 ± 0e	10 ± 8a	81 ± 30a

For each level of days of treatment under each genotype, the significant differences between means are shown with different letters at P < 0.05.

### 3.2 Effect of brassinolide on stored substances in Korean pine embryogenic calli

#### 3.2.1 Effect of brassinolide on soluble sugar content

**Figure 2:**
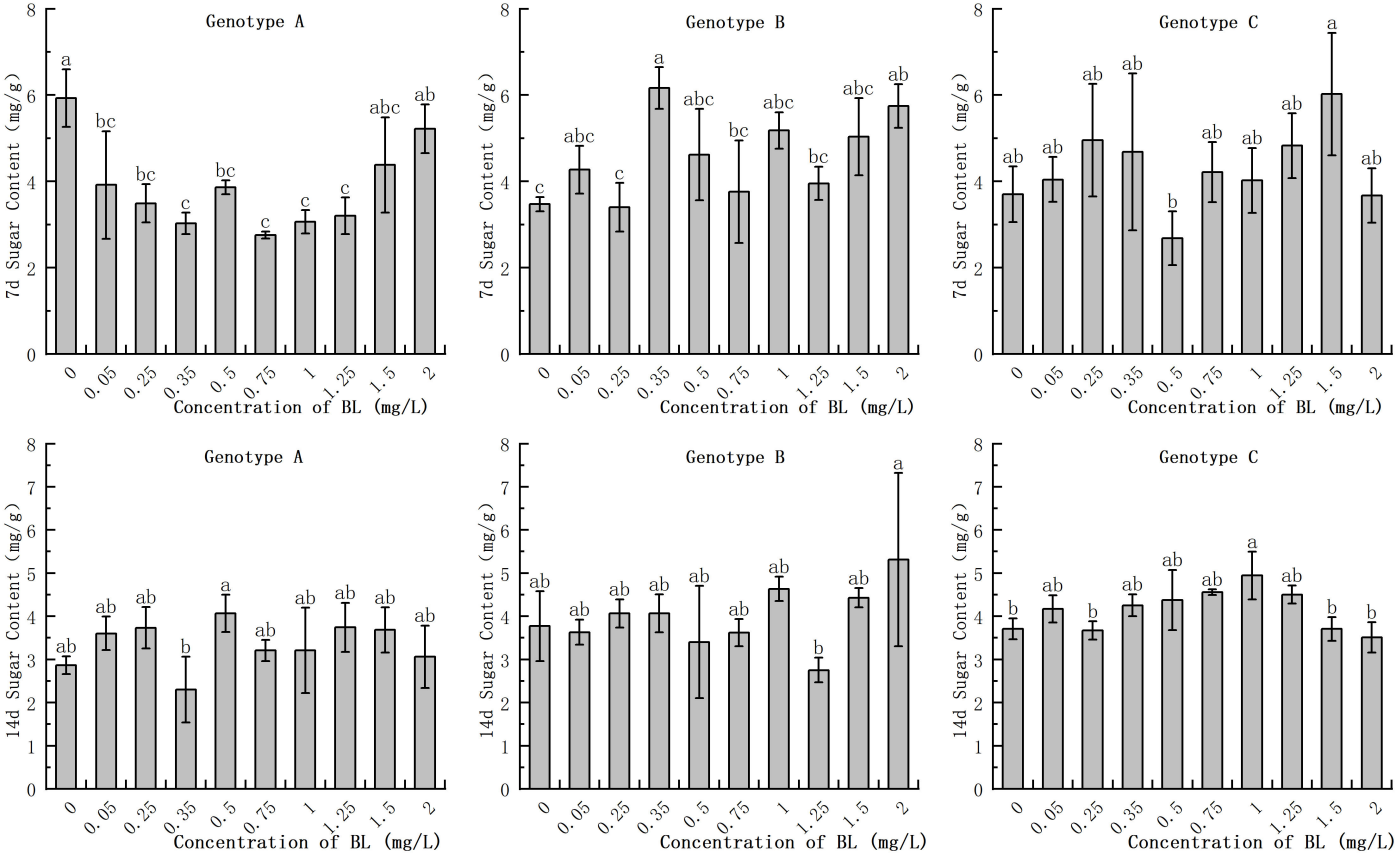
Effects of brassinolide on soluble sugar content during growth in embryogenic calli of Korean pine. For each concentration level of treatment at each genotype, the significant differences between the means with different letters are shown at *P < 0.05*.

The embryogenic callus of genotype A had a higher soluble sugar content in the early stages compared than that of genotypes B and C. However, this content decreased with increasing progeny. However, this content decreased only slightly with increasing proliferation time increased. When genotype A was cultured with BL for 7 days, the experimental groups, showed a significant decrease in soluble sugar content, with the exception of 1.50 mg/L and 2.00 mg/L (**Figure 2**). After 14 days of culture, the soluble sugar concentration decreased significantly in the experimental group at 0.35 mg/L experimental group decreased significantly, but there was no significant difference between the other treatments and the control. In genotype B, the addition of brassinolide for 7 days resulted in a significant increase in soluble sugar content in the 0.35 mg/L and 2.00 mg/L treatment groups, with a content of 6.17 mg/g and 5.75 mg/g, respectively (**Figure 2**). However, the difference between the content of the experimental and control groups was no longer significant after 14 days of proliferation culture. The soluble sugar content in the genotype C cells did not differ significantly with the increasing culture duration of the brassinolides (**Figure 2**).

#### 3.2.2 Effect of brassinolide on starch content

**Figure 3:**
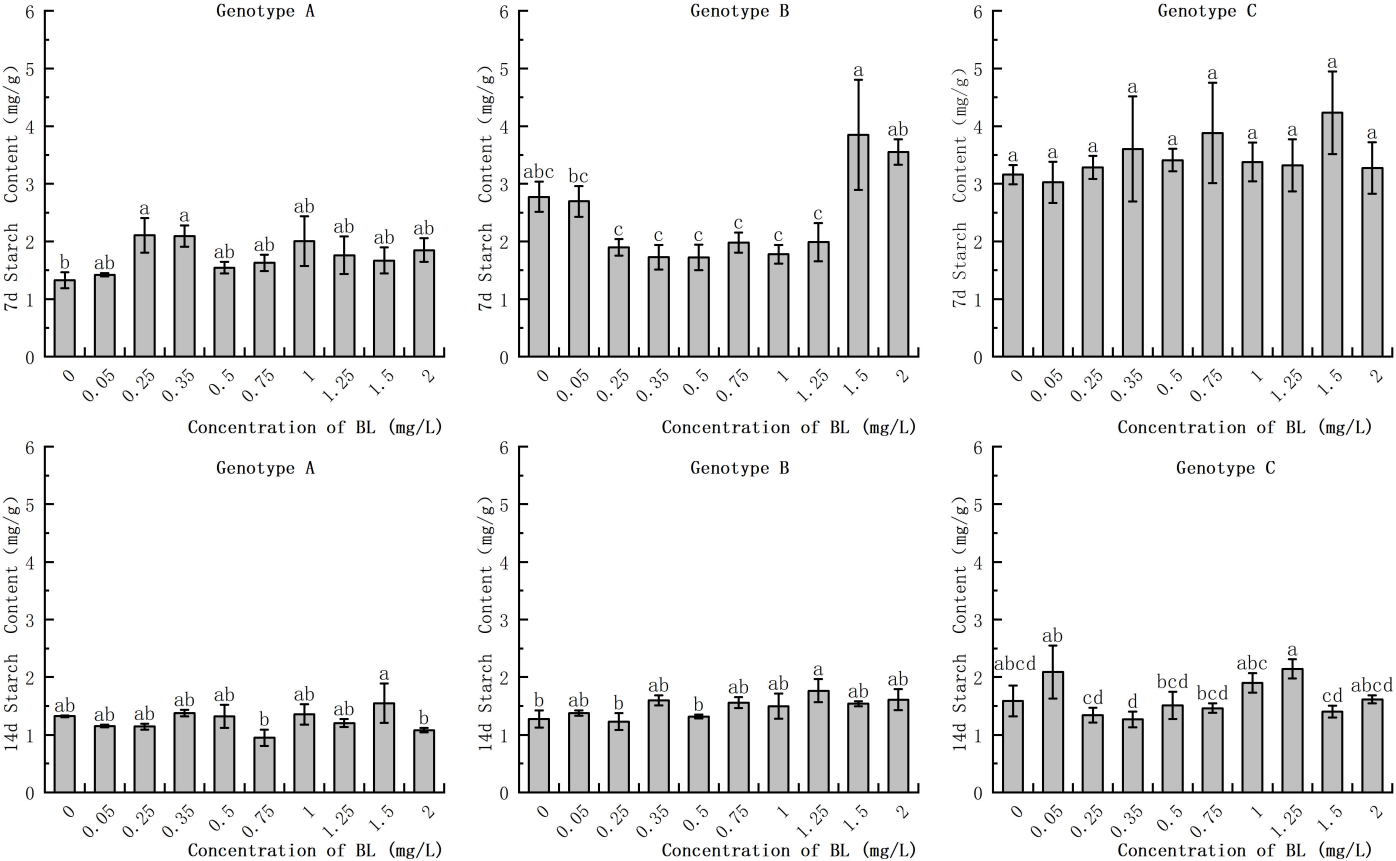
Effects of brassinolide on starch content during growth in Korean pine embryogenic callus. For each level of treatment level and genotype, the significant differences between the means are shown with different letters *P < 0.05*.

In the embryogenic callus of genotype A, the starch content in the experimental groups with 0.25 mg/L and 0.35 mg/L was significantly higher at 2.11 mg/g and 2.09 mg/g respectively, than in the control group which had a starch content of 1.33 mg/g (**Figure 3**). With increasing cultivation time, the starch content in the experimental group of genotype A decreased to varying degrees, while no significant difference was found in the starch content of the control group. In the embryogenic callus of genotype B, there was no significant difference in starch content between the experimental group and the control group after 7 days of brassinolide addition (**Figure 3**). After 14 days of culture, the starch content decreased significantly in both the experimental and control groups decreased significantly, but there was no significant difference between the concentration gradients. In the embryogenic callus of genotype C, the starch content was significantly lower after 14 days of culture than after 7 days. However, the difference between the starch content of the different concentration gradients at each stage was not significant (**Figure 3**).

#### 3.2.3 Effect of brassinolide on soluble protein content

**Figure 4:**
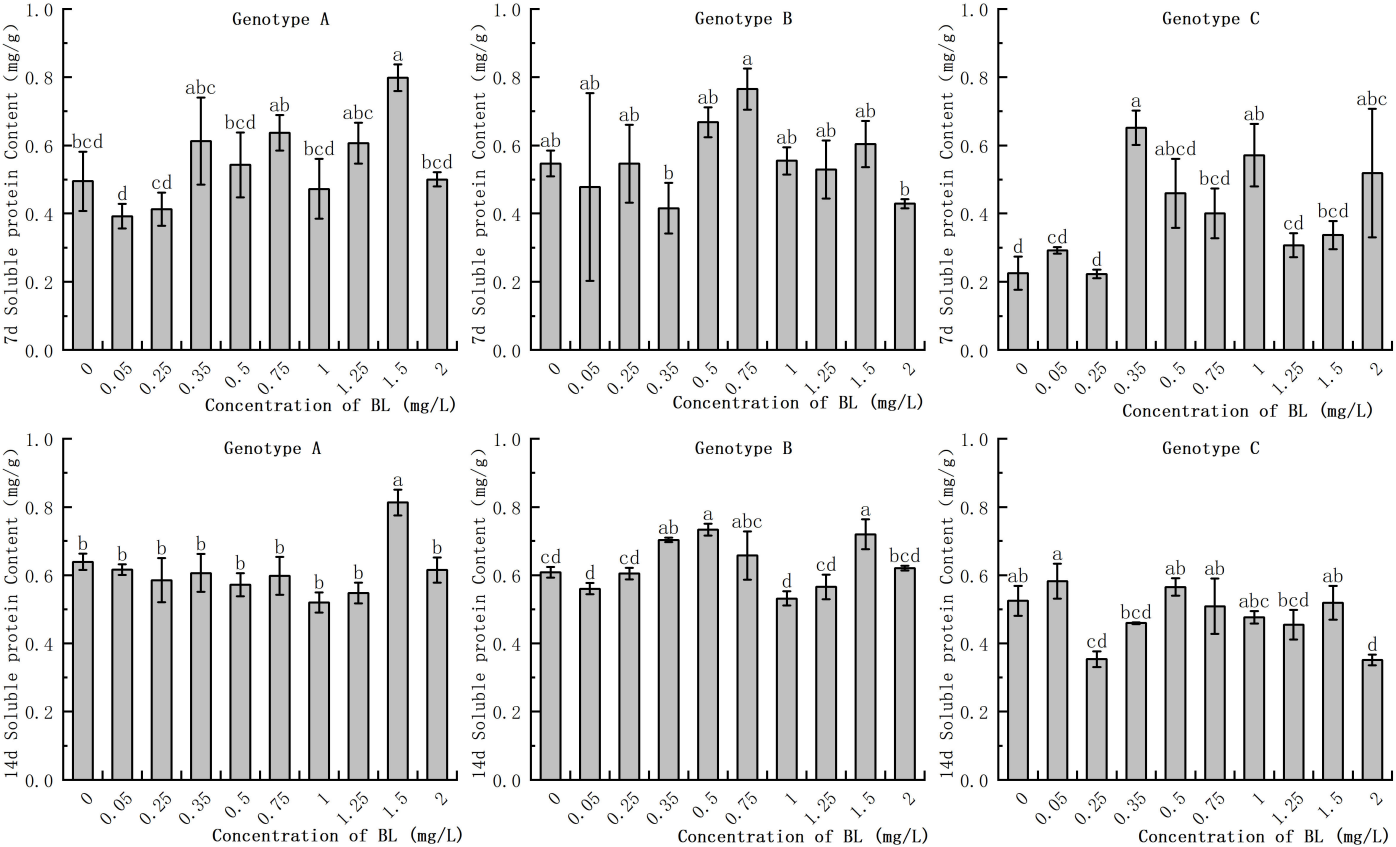
Effects of brassinolide on starch content during growth in Korean pine embryogenic callus. For each level of treatment under each genotype, the significant differences between the means are shown with different letters *P < 0.05*.

In the embryogenic callus of genotype A, the soluble protein content in the control group increased significantly as with increasing culture duration. However, no significant differences were observed in the experimental group with prolonged culture time. At 1.50 mg/L, the soluble protein content was significantly higher than that of the control group in the same period, with values of 163.27% and 126.56% of the control group after 7 days and 14 days, respectively (**Figure 4**). In the embryogenic callus of genotype B, the accumulation of soluble protein in the cells decreased significantly with increasing culture time. There was no significant difference in soluble protein content between the experimental group and the control group during the 7 day treatment, but the content was significantly higher at 0.35 mg/L in the 14 day treatment. At concentrations of 0.35 mg/L, 0.5 mg/L, and 1.5 mg/L, the soluble protein contents were 0.7 mg/g, 0.73 mg/g, and 0.72 mg/g, respectively, which were significantly higher than those of the control group in the genotype B embryogenic callus. In the embryogenic callus of genotype C, the soluble protein content after 7 days of treatment was significantly higher than in the control group at concentrations of 0.35 mg/L, 1.00 mg/L, and 2.0 mg/L, with values of 0.65 mg/g, 0.57 mg/g, and 0.52 mg/g, respectively (**Figure 4**). The total soluble protein content in the embryogenic callus of genotype C was significantly increased when cultured for 14 days. The soluble protein content was significantly lower than that of the control group at 0.25 mg/L and 2.0 mg/L, while the other concentrations were similar to those of the control group and showed no significant difference (**Figure 4**).

### 3.3 Effect of brassinolide on MDA content in embryogenic callus of Korean pine

**Figure 5:**
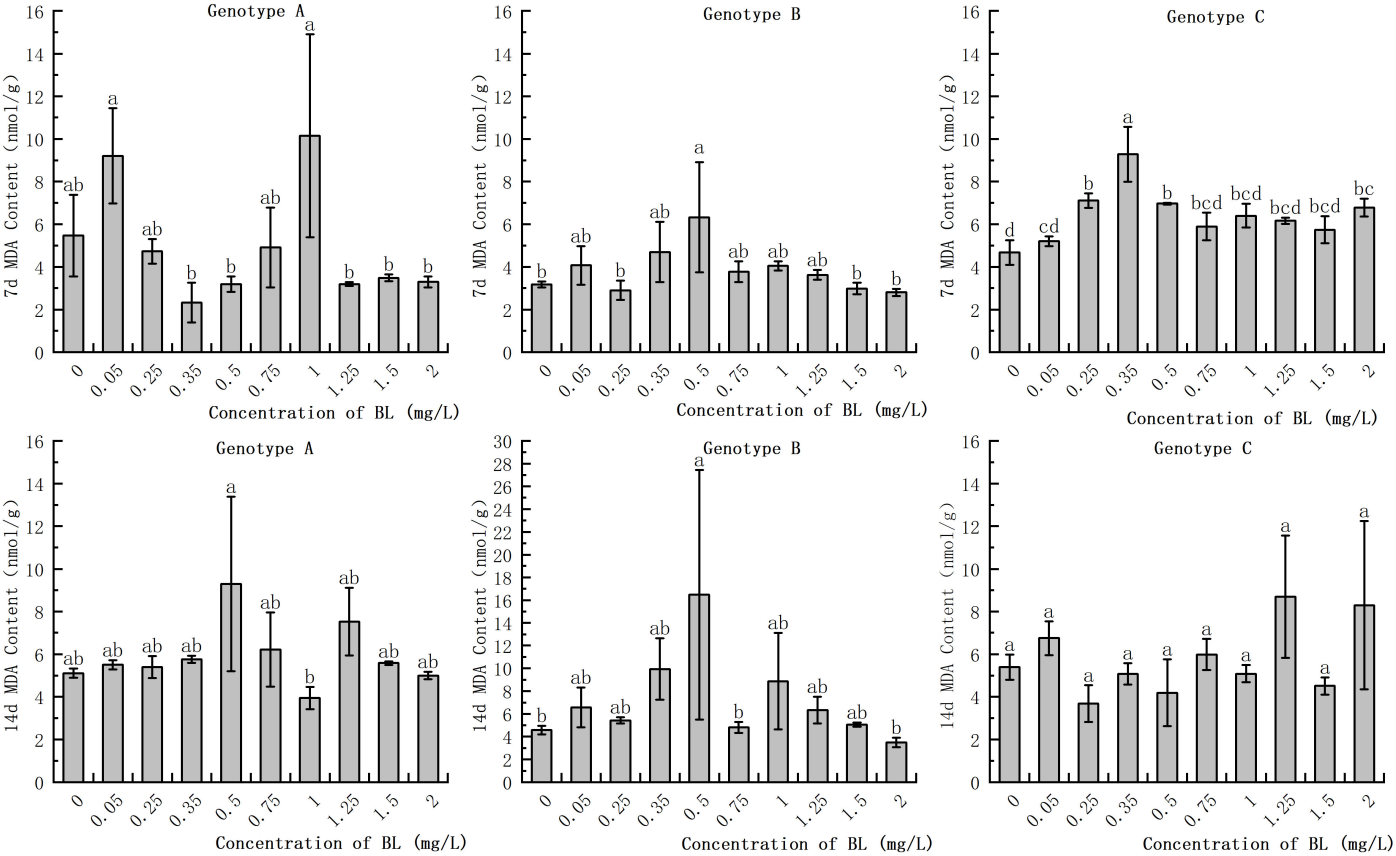
Effects of brassinolide on MDA content during growth in Korean pine embryogenic callus. For each treatment level and each genotype, the significant differences between the mean values are shown with different letters at *P < 0.05*.

The study showed that there was no significant difference in MDA levels in the embryogenic callus of genotype A after treatment with different concentrations of brassinolide. However, at concentrations of 0.05 mg/L and 1.00 mg/L, MDA activities increased by 68.19% and 85.37%, respectively, while at concentrations of 0.50 mg/L and 1.25 mg/L, MDA increased by 81.80% and 47.36%, respectively, after14 days of proliferation, but the difference was not significant (**Figure 5**). In genotype B, the MDA activity at 0.5 mg/L was significantly higher than that of the control group, with an increase of 199.37% and 360.61% after 7 and 14 days, respectively (**Figure 5**). In genotype C, the MDA activity after cultivation with 0.35 mg/L brassinolide was 149.25% of that of the control group, but the difference was not significant compared to the control group (**Figure 5**).

### 3.4 Effect of brassinolide on antioxidant enzyme activity in embryogenic callus of Korean pine

#### 3.4.1 Effect of brassinolide on SOD activity

**Figure 6:**
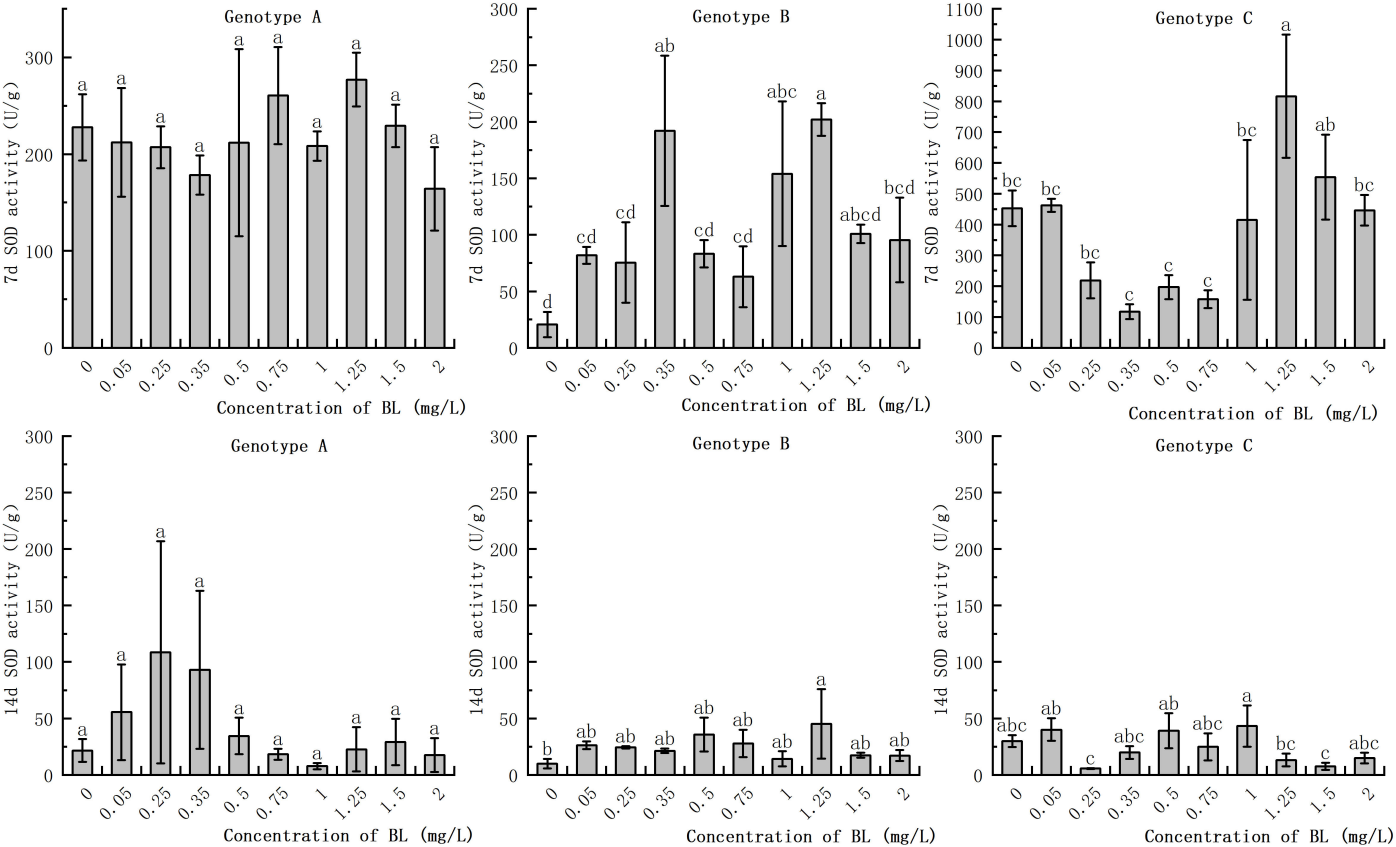
Effects of brassinolide on the Activity of SOD during growth in Korean pine embryogenic callus. For each treatment level and genotype, the significant differences between the mean values are shown with different letters *P < 0.05*.

In the embryogenic callus of the three genotypes, SOD activity decreased significantly with increasing in culture duration. The range of reduction was extremely wide. A partial concentration of brassinolide in the embryogenic callus of genotype A had hardly any effect on SOD activity at the same proliferation time, with no significant difference (**Figure 6**). In genotype B, SOD activities at concentrations of 0.35 mg/L, 1.00 mg/L, and 1.25 mg/L were 9.33% and 7.48%, respectively, of those of the control group when BL was added for 7 days. However, at a concentration of 1.25 mg/L, the SOD activity was 9.81 times higher than that of the control group. After 14 days of culture, the SOD activity in the experimental group increased by 354.93% compared to that in the control group (**Figure 6**). The SOD activity of the embryogenic callus of genotype C was the highest among the three genotypes after 7 days, reaching 816.37 U/g at concentrations of 1.25 mg/L and 1.50 mg/L and 553.82 U/g, which were significantly higher than that of the control group (**Figure 6**).

#### 3.4.2 Effect of brassinolide on POD activity

**Figure 7:**
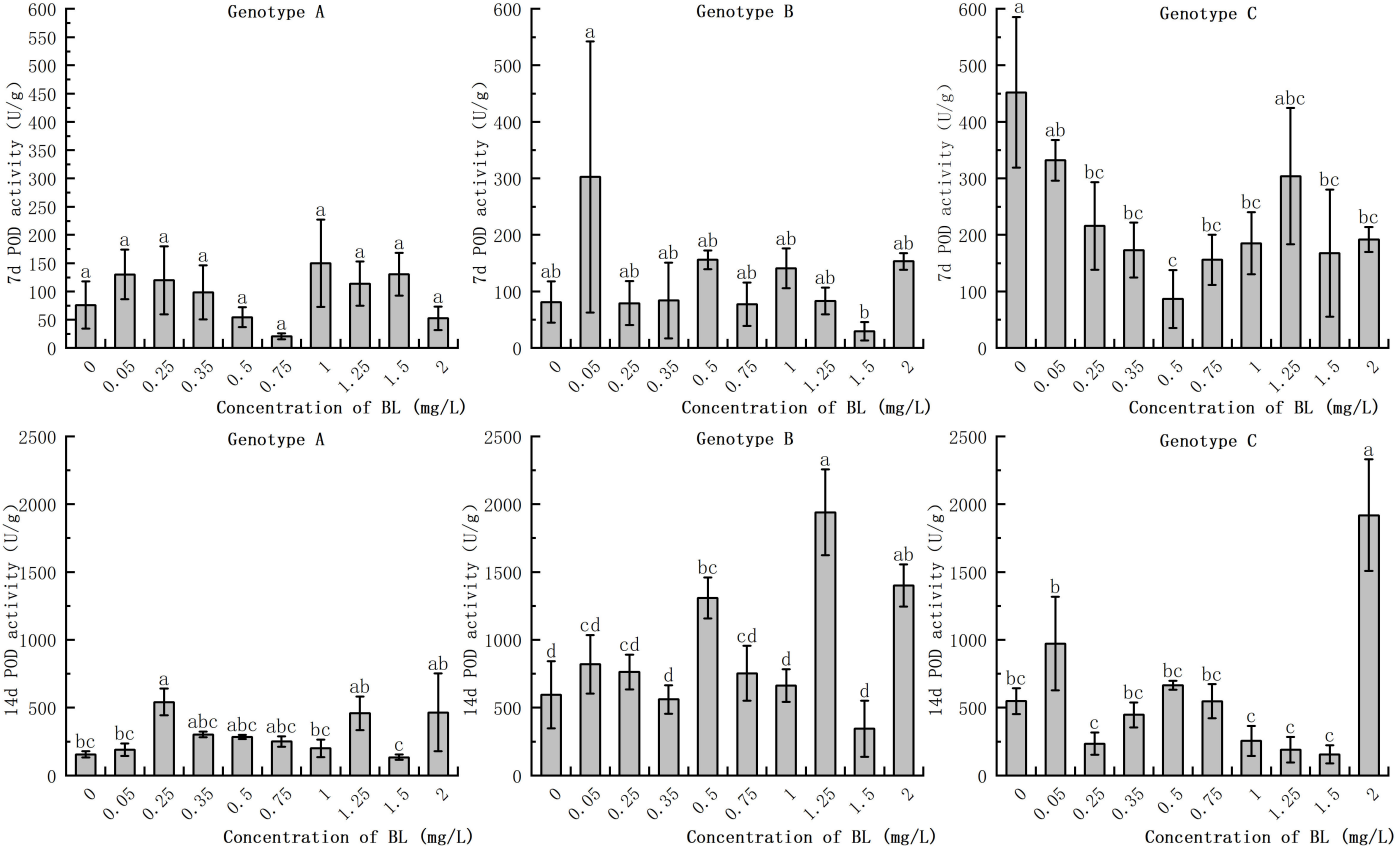
Effects of brassinolide on POD activity during growth in Korean pine embryogenic callus. For each treatment level and genotype, the significant differences between the mean values are shown with different letters *P < 0.05*.

POD activity increased to a greater extent in all three genotypes with increasing proliferation time, although the extent of the increase differed considerably. While the POD activity of the embryogenic callus in the experimental group increased slightly in some treatments after the addition of brassinolide to the embryogenic callus of genotype A, the difference was not significant compared to the control group (**Figure 7**). On the 14th day after the addition of brassinolide, the POD activity of the embryogenic callus treated with 0.25 mg/L brassinolide increased by 34.38% compared to the control group, and the difference was significant. Genotype B embryogenic callus of genotype B showed no significant difference in POD activity between the experimental groups after being cultured with brassinolide for 7 days. However, after adding brassinolide and culturing for 14 days, POD activity at the concentrations of 0.50 mg/L, 1.25 mg/L, and 2.00 mg/L increased by 119.95%, 226.23%, and 135.42% respectively, compared with the control group, respectively, and the difference was significant (**Figure 7**). Although the POD activity increased significantly at other concentrations, the difference was not significant compared to the control group. When the embryogenic callus of genotype C was cultured with brassinolide for 7 days, the POD activity decreased significantly compared with the control group. The POD activity after 0.5 mg/L culture was only 19.03% of the control group. After 14 days of culture with brassinolide, the POD activity of the experimental group was significantly higher than that of the control group, with the POD activity after 2.00 mg/L culture was 349.88% of the control group. However, the POD activity of the other groups remained relatively stable and showed no significant differences after culture (**Figure 7**).

#### 3.4.3 Effect of brassinolide on CAT activity

**Figure 8:**
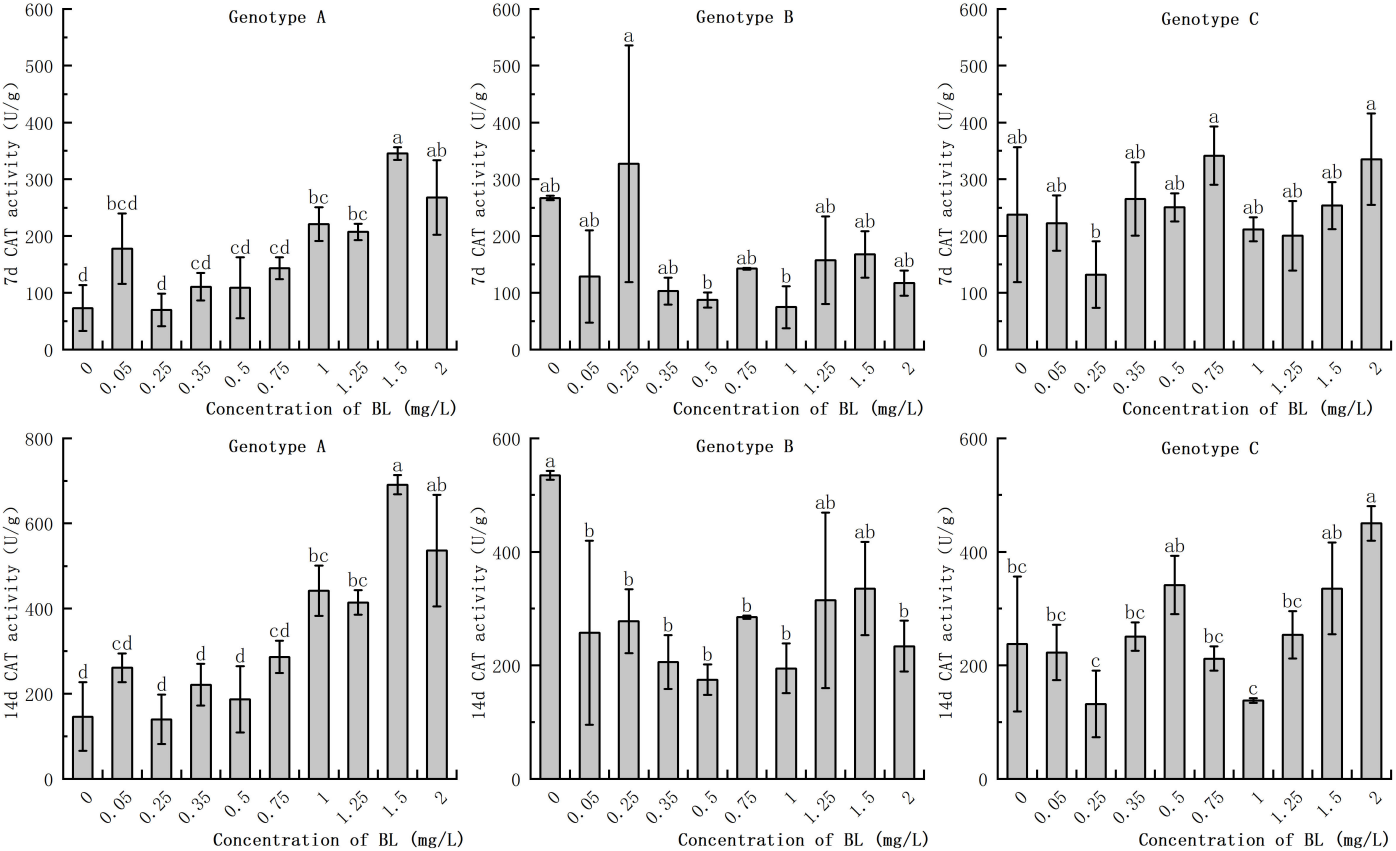
Effects of brassinolide on the activity of CAT during growth in Korean pine embryogenic callus. For each treatment level and genotype, the significant differences between the means are shown with different letters *P < 0.05*.

The CAT activity of the embryogenic callus in the genotypes increased with the prolongation of the proliferation time. However, there was no significant difference in CAT activity between the embryogenic callus of genotype A and genotype C with proliferation time. When the embryogenic callus of genotype A was cultured with brassinolide, the CAT activity was significantly higher in the experimental group with a concentration of more than 0.75 mg/L was significantly higher than in the control group. After 7 days, the CAT activity in the experimental group was 4.72 times higher than in the control group, and after 14 days, it was 4.71 times higher (**Figure 8**). The CAT activity of the embryogenic callus of genotype B did not differ significantly from that of the control group after 7 days of culture with brassinolide. After 14 days of proliferation and culture, the CAT activity in the experimental group at 0.50 mg/L was only 32.70% of that of the control group (**Figure 8**). When brassinolide was added to the embryogenic callus of genotype C and cultured for 7 days, the intracellular CAT activity increased, but the difference was not significant. The CAT activity of the other experimental groups, with the exception of 1.25 mg/L and 1.50 mg/L, was not significantly different from that of the control group. The CAT activity after treatment with 2.00 mg/L was 189.43% higher than that of the control group (**Figure 8**).

### 3.5 Analysis of the correlation between physiological indicators of embryogenic callus and maturation transition in somatic embryos

**Figure 9:**
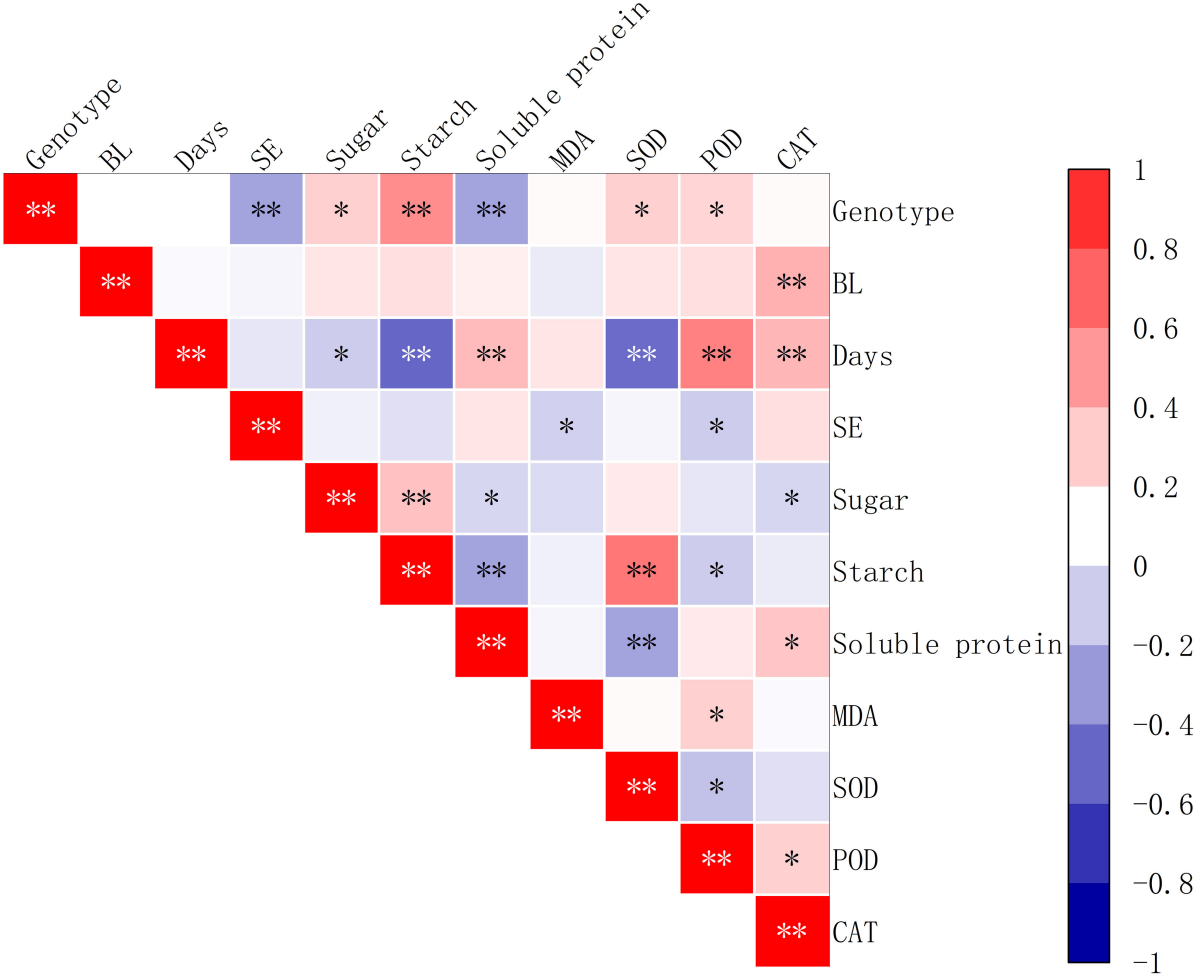
Correlation between the yield of somatic embryos and physiological indicators after the addition of brassinolides. SE stands for the number of embryogenic transformations, and BL for brassinolide in the embryogenic callus. Asterisks indicate significant differences, *P < 0.5 and **P < 0.01.

The study showed that the addition of brassinolide at different concentrations had a positive correlation with the activity of CAT (*P<0.01*). It was also found that, the duration of culture time for the addition of brassinolide had a significant positive correlation with soluble protein content, POD activity, and CAT activity (*P<0.01*). However, it was found that the cultivation period for the addition of brassinolide had a significant negative correlation with starch content and SOD activity (P<0.01), as well as soluble sugar content (*P<0.5*). It was also found that somatic embryo yield was significantly negatively correlated with MDA content and POD activity (*P<0.05*) (**Figure 9**).

### 3.6 Effect of brassinolide on key hormones for preservation of embryogenic callus of Korean pine

#### 3.6.1 Effect of brassinolide on IAA content

**Figure 10:**
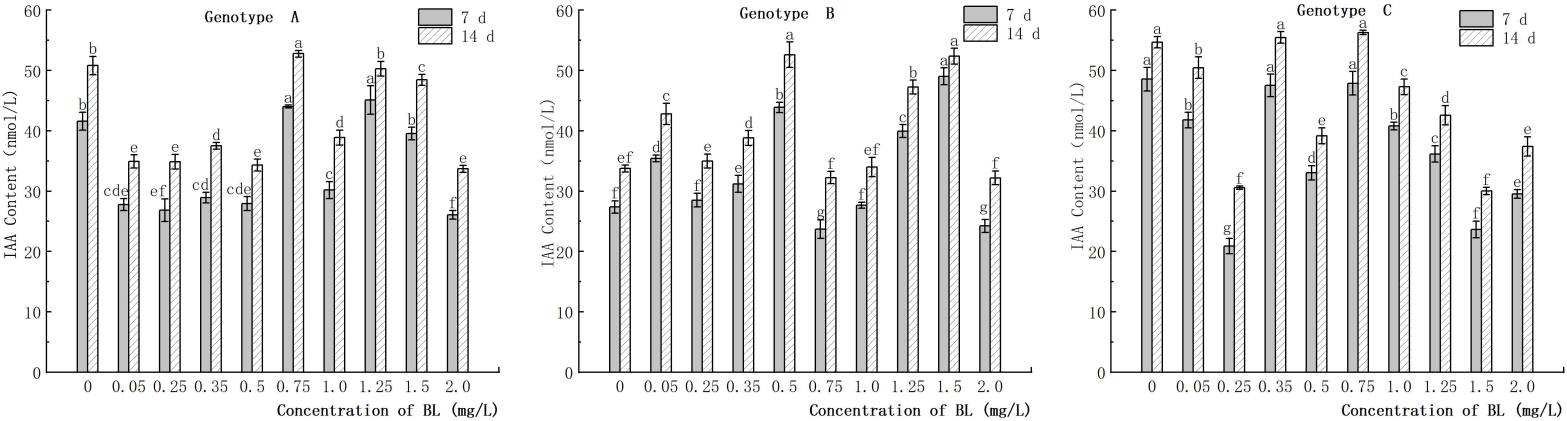
Effects of brassinolide on IAA content during growth in Korean pine embryogenic callus. For each treatment level under each genotype, the significant differences between the means are shown with different letters at *P < 0.05*.

The endogenous IAA content in the embryogenic callus of genotype A decreased significantly when cultured with different concentrations of brassinolide, except for the concentrations of 0.75 mg/L, 1.25 mg/L, and 1.50 mg/L. The IAA content in the experimental groups with concentrations lower than 0.50 mg/L was generally lower than in the control group, but there was no significant difference between the treatment concentrations (**Figure 10**). In the embryogenic callus of genotype B, the endogenous IAA content increased significantly compared to that of the control group when cultured with different concentrations of brassinolide. The IAA concentration in the experimental group increased by 29.49%, 60.34%, 46.05%, and 79.17% in the 7-day culture and by 26.81%, 55.91%, and 40.00% in the 14-day culture at concentrations of 0.05 mg/L, 0.50 mg/L, 1.25 mg/L, and 1.50 mg/L concentrations, respectively. The difference was significant at 55.17% (**Figure 10**). After the embryogenic callus of genotype C was cultured with different concentrations of brassinolide, the endogenous IAA content showed a downward trend with increasing brassinolide concentration. The IAA content decreased significantly at concentrations of 0.25 mg/L, 0.50 mg/L, and 1.50 mg/L compared to that of the control group. At a brassinolide concentration of 0.25 mg/L, the IAA content was only 43.07% (7 d) and 55.93% (14 d) of the control group, and the difference was significant compared to the control group during the same period (**Figure 10**).

#### 3.6.2 Effect of brassinolide on ABA content

**Figure 11:**
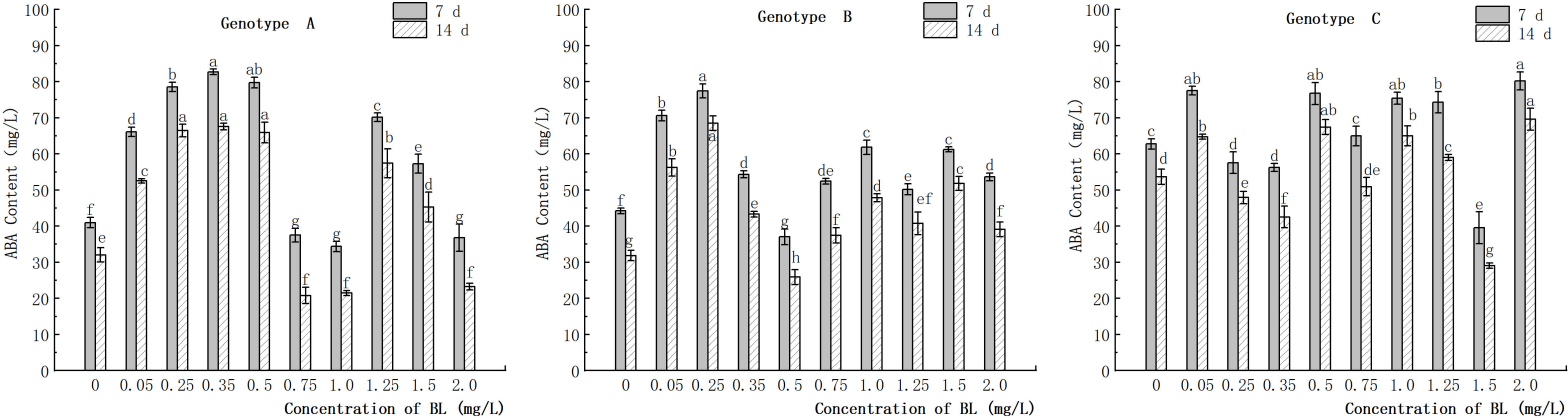
Effects of brassinolide on ABA content during growth in Korean pine embryogenic callus. For each treatment level and genotype, the significant differences between the means are shown with different letters *P < 0.05*.

The study showed that the embryogenic callus of genotype A exhibited a significant increase in endogenous ABA content with an increase in brassinolide concentration up to 0.5 mg/L (**Figure 11**). However, at 0.75 mg/L and 1.0 mg/L, the ABA content in the experimental groups was significantly lower than in the control group. Specifically, the ABA content was 37.49 ng/g and 34.38 ng/g after 7 days of brassinolide culture and 20.78 ng/g and 21.47 ng/g, after 14 days, respectively. In contrast, the endogenous ABA content in the treatment group was significantly higher than in the control group at more than 1.25 mg/L, but the ABA content decreased significantly with increasing concentration (**Figure 11**). In genotype B, the content of endogenous ABA initially increased in the experimental group, then decreased, and then increased again with increasing brassinolide concentration. The highest value was observed at a concentration of 0.25 mg/L (77.43 ng/g after 7 days, 68.51 ng/g after 14 days), and the lowest value at a concentration of 0.50 mg/L (37.06 ng/g after 7 days, 25.91 ng/g after 14 days). These differences were significant compared to the control group. After culturing the embryogenic callus of genotype C with different concentrations of brassinolide, it was found that the endogenous ABA content was slightly higher in the experimental group than in the control group in most treatments with brassinolide concentrations. This difference was statistically significant (**Figure 11**). The increase in endogenous ABA content in the experimental group after treatment with each concentration was within 25.0% of that in the control group. However, the endogenous ABA content in the experimental group with a brassinolide concentration of 1.50 mg/L was significantly lower than that in the control group during the same period. It decreased by 36.92% and 45.83% on the 7th and 14th day of culture, respectively (**Figure 11**).

### 3.7 Effect of brassinolide on endogenous BR content in preservation of embryogenic callus of Korean pine

**Figure 12:**
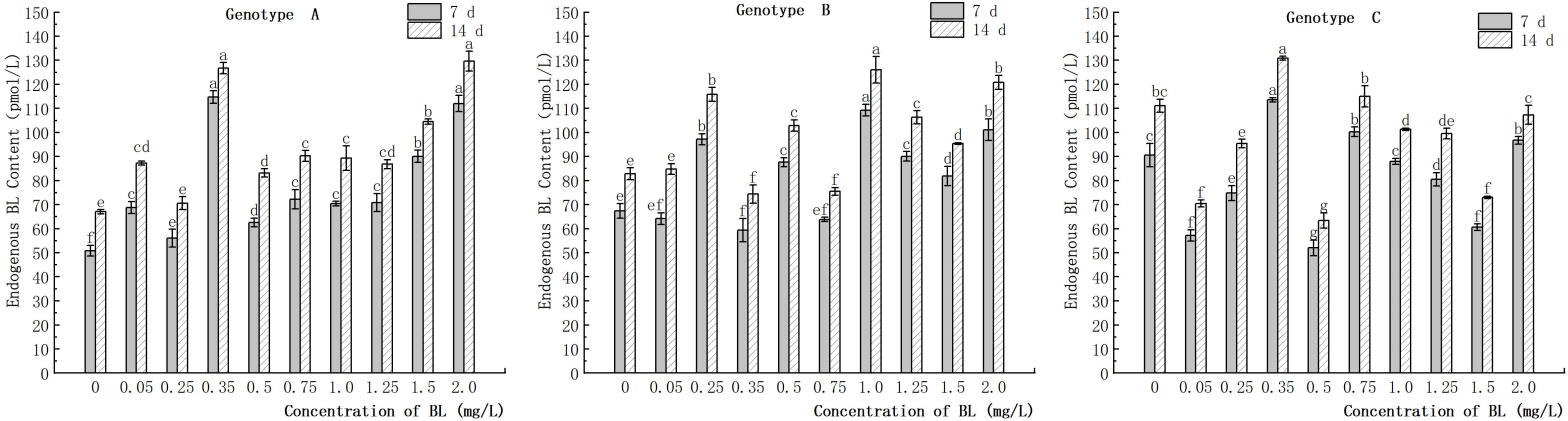
Effects of brassinolide on endogenous BL content during in Korean pine embryogenic callus. For each treatment level and genotype, the significant differences between the means are shown with different letters *P < 0.05*.

The study showed that the content of endogenous BL in the embryogenic callus of genotype A increased with the concentration of brassinolide, with the highest increase observed at 0.35 mg/L. At concentrations of more than 1.25 mg/L, the rate of increase in endogenous BL content was higher than that in the other experimental groups (**Figure 12**). In genotype B, the content of endogenous BL content also increased with the concentration of brassinolide, with the highest increase observed at 0.25 mg/L and 1.00 mg/L (**Figure 12**). In genotype C, however, the endogenous BL content decreased in most experimental groups after the addition of exogenous brassinolide. In particular, at 0.05 mg/L, 0.50 mg/L, and 1.50 mg/L, the endogenous BL content decreased by 36.91%, 42.62% and 33.11%, respectively, after 7 days of treatment compared to the control. The decrease continued after 14 days of treatment, with decreases of 36.55%, 39.34% and 36.99%, respectively (**Figure 12**). These differences were significant.

### 3.8 Effect of brassinolide on PAL activity in embryogenic callus of Korean pine

**Figure 13:**
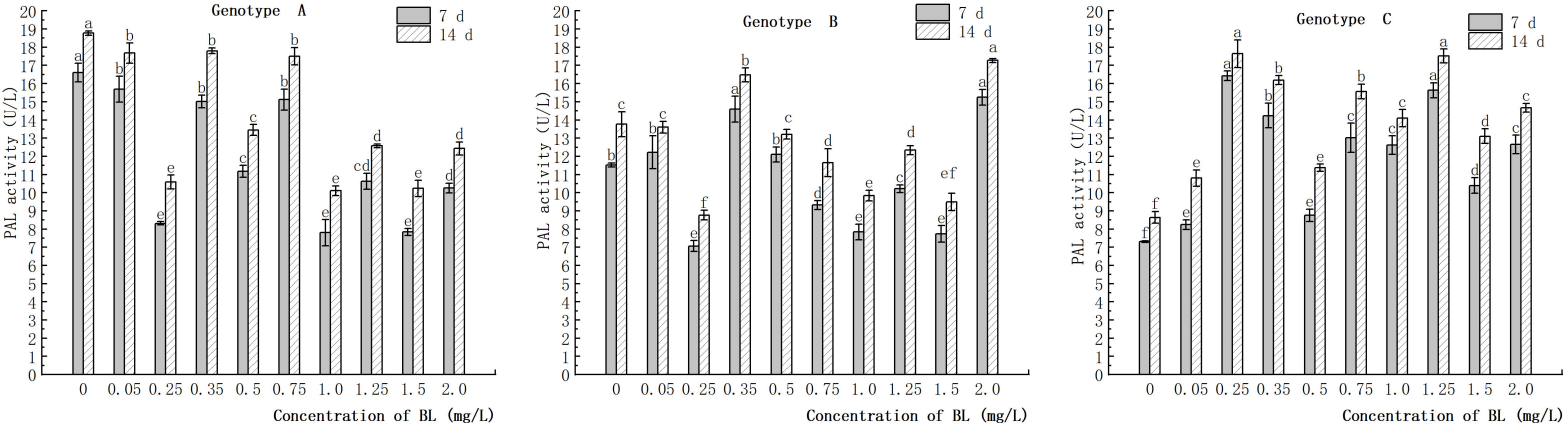
Effects of brassinolide on PAL activity during growth in Korean pine embryogenic callus. For each treatment level of at each genotype, the significant differences between the means with different letters are shown at *P < 0.05*.

The study showed that the embryogenic callus of genotype A exhibited a decrease in endogenous PAL activity with an increase in brassinolide concentration. However, this decrease was attenuated after treatment with certain concentrations (0.35 mg/L, 0.75 mg/L, and 2.00 mg/L). The rate of decline was significantly lower than that of the control group. In genotype B, the embryogenic callus also showed a decrease in endogenous PAL activity with an increase in brassinolide concentration, except for 0.35 mg/L and 2.0 mg/L. PAL activity decreased rapidly after 0.25 mg/L culture, and was 7.06 U/L (7 d) and 8.76 U/L (14 d), which was significantly different from the control group. Compared to the control group, the experimental group with embryogenic callus of genotype C showed a significant increase in PAL activity to varying degrees. The highest PAL activity values were observed at 0.25 mg/L and 1.25 mg/L. After 7 and 14 days of brassinolide treatment, PAL activities of 16.42 U/L, 15.63 U/L, 17.64 U/L and 15.63 U/L, were observed. The value of PAL activity value after 14 days of brassinolide treatment was 17.52 U/L (**Figure 13**).

### 3.9 Effect of brassinolide on content of lignin in embryogenic callus of Korean pine

**Figure 14:**
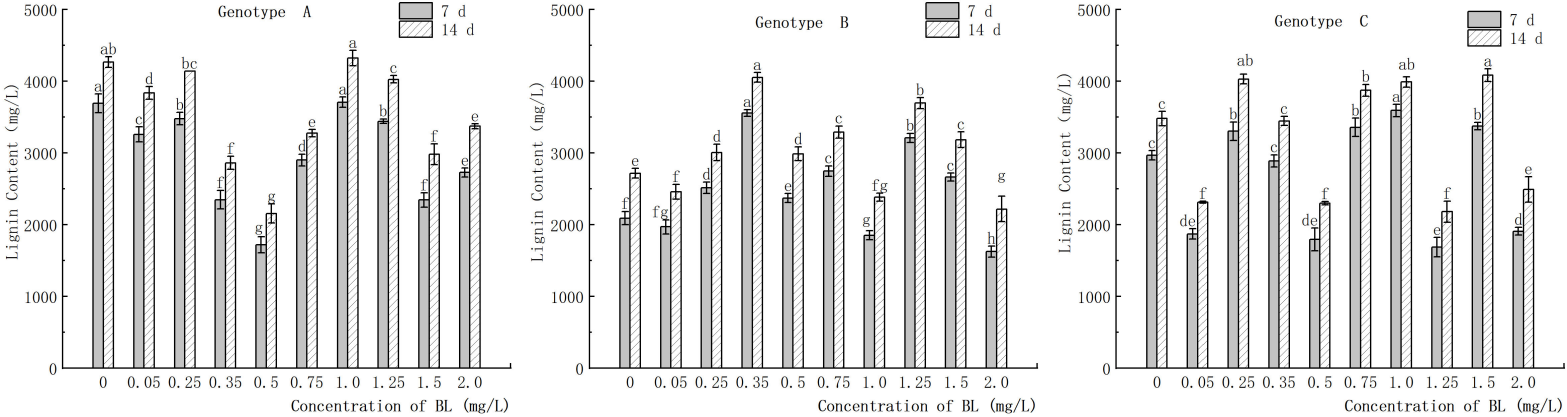
Effects of brassinolide on lignin content during growth in embryogenic callus of Korean pine. For each treatment level and genotype, the significant differences between the mean values are shown with different letters *P < 0.05*.

The study showed that in genotype A, the endogenous lignin content in the embryogenic callus was significantly lower in the experimental group than in the control group, except at 0.25 mg/L and 1.00 mg/L (**Figure 14**). After the addition of brassinolide, the lignin content initially showed a decreasing trend, followed by an increase and then a decrease again with increasing brassinolide concentration. The lowest point was observed at a concentration of 0.50 mg/L, and the activity decreased after 7 days of culture (**Figure 14**). The embryogenic callus of genotype A showed an activity of 1718.24 ng/g, which increased to 2153.71 ng/g after 14 days of culture. In genotype B, the highest lignin content was observed at brassinolide concentrations of 0.35 mg/L and 1.25 mg/L, with 3553.94 ng/g after 7 days of culture and 3208.14 ng/g after 14 days of culture (**Figure 14**). The lignin content was 4050.85 ng/g and 3692.13 ng/g after 14 days of culture. After treatment with brassinolide, the lignin content of some concentration gradients was significantly reduced compared to the control, with brassinolide concentrations of 0.05 mg/L, 0.50 mg/L, 1.25 mg/L, and 2.00 mg/L causing the lignin content to decrease to about 2000 ng/g after 7 days of culture and below 2500 ng/g after 14 days of culture, which was significantly different from the control.

### 3.10 Change in IAA/ABA ratio in the embryogenic callus of Korean pine after brassinolide addition

When the embryogenic callus of genotypes A and B were treated with brassinolide concentrations of less than 1.0 mg/L, the intracellular concentrations of IAA and ABA showed an inverse relationship. When 0.75 mg/L brassinolide was added to genotype A, the IAA content increased, while the ABA content decreased. Conversely, when the brassinolide concentration was less than 0.75 mg L^-1^, the ABA content increased, and the IAA content decreased (**Figures 10**, **11**). The same trend was observed in genotype B, where a concentration of 0.25 mg/L brassinolide resulted in a higher ABA content and a lower IAA content, while a concentration of 0.50 mg/L brassinolide resulted in a higher IAA content and a lower ABA content (**Figures 10**, **11**). In genotype C, the addition of 0.35 mg/L and 0.75 mg/L brassinolide to the embryogenic callus resulted in a high IAA content and a low ABA content, while the addition of 0.50 mg/L brassinolide resulted in an opposite ratio between the two (**Figures 10**, **11**).

**Figure 15:**
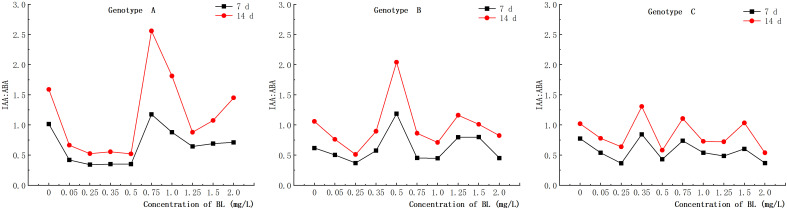
Effects of brassinolide on the IAA/ABA ratio during growth in embryogenic Callus of Korean pine.

The addition of different amounts of brassinolide to the embryogenic callus of genotypes A, B and C resulted in different levels of endogenous IAA: ABA ratios. In genotype A, the highest peak endogenous IAA: ABA ratio was observed after the addition of 0.75 mg/L brassinolide, which was significantly higher than that of the control group (**Figure 15**). Similarly, in genotype B, the highest peak of endogenous IAA: ABA ratio was observed after the addition of 0.50 mg/L brassinolide, which was significantly higher than that of the control group (**Figure 15**). On the other hand, in genotype C, the highest peak endogenous IAA: ABA ratio was observed after the addition of 0.35 mg/L, 0.75 mg/L, and 1.50 mg/L brassinolide, which were all significantly higher than that of the control group (**Figure 15**).

### 3.11 Analysis of the correlation between endogenous hormones, PAL activity, and lignin content of embryogenic callus and maturation transition in somatic embryos

**Figure 16:**
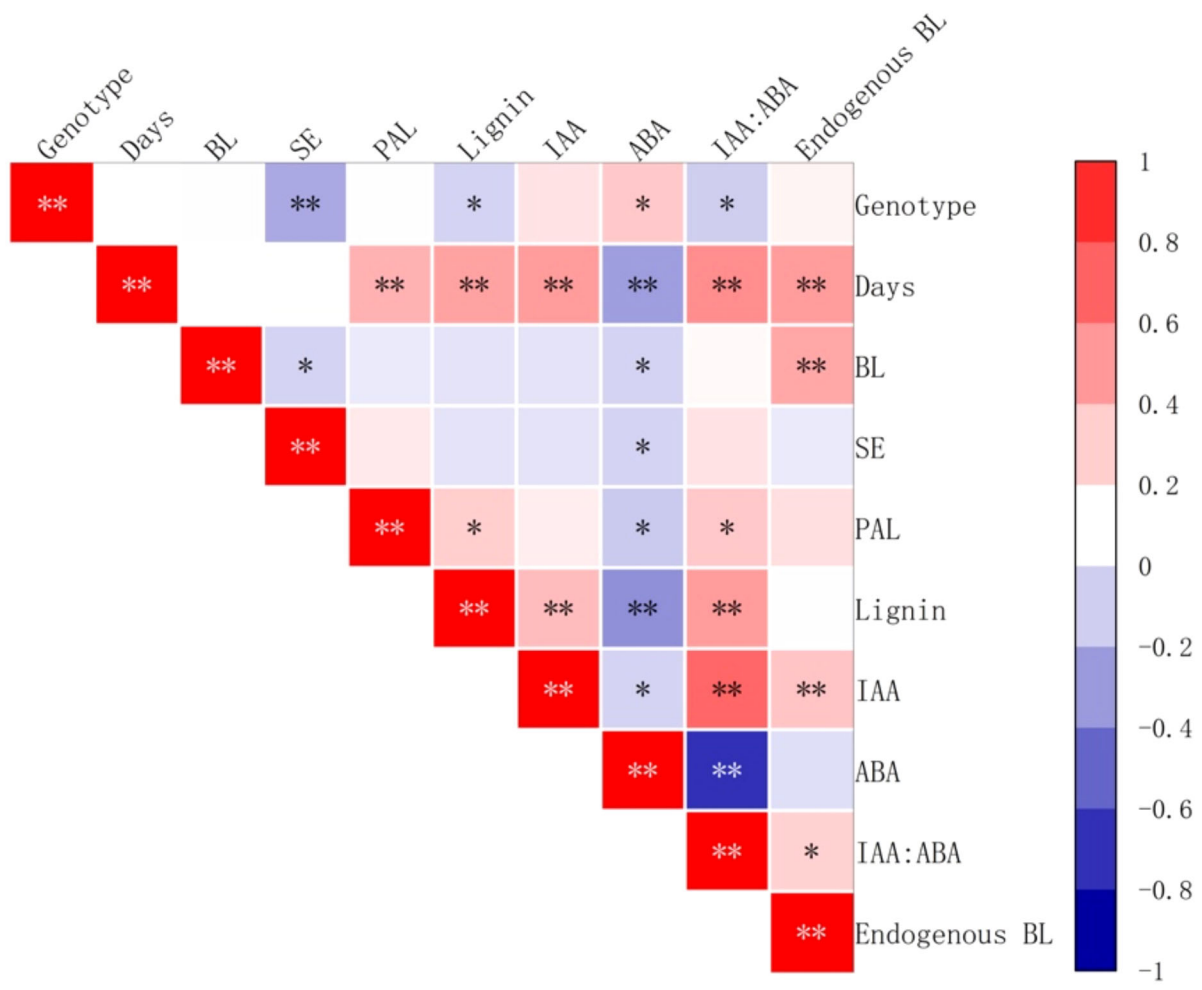
Correlation between the yield of somatic embryos and physiological indicators after brassinolide the addition of brassinolides. SE stands for the number of embryogenic transformations and BL for BL in embryogenic callus. Asterisks indicate significant differences, *P < 0.5 and **P < 0.01.

The addition of brassinolide during the culture period had a significant positive correlation with PAL activity, lignin content, IAA content, and IAA:ABA ratio (*P < 0.01*) (**Figure 16**). Endogenous BL content also showed a significant positive correlation *(P < 0.01*) (**Figure 16**). On the other hand, there was a significant negative correlation (*P < 0.01*) (**Figure 16**) between ABA content and the above-mentioned factors. In addition, the brassinolide concentration and endogenous ABA content is significantly negatively correlated with the number of somatic embryo transfers (*P < 0.5*) (**Figure 16**).

## 4 Discussion

### 4.1 Effect of brassinolide on the number of embryogenic embryos converted in calluses

The study showed that with increasing duration of culture, the growth rate of non-embryogenic callus exceeded that of embryogenic callus, leading to an overall decrease in the embryogenic quality of the callus. In particular, for genotype A, which exhibited weak embryogenicity, the addition of a higher concentration (0.75 mg/L) of brassinolide to the culture medium over a 14-day period resulted in a significant 1.6 times increase in growth rate. In the case of genotype B, which exhibited strong embryogenicity, the addition of 0.05 mg/L brassinolide and a 7-day culture period led to a significant increase in the number of somatic embryo conversions, which reached about 2.3 times that of the control group. When 0.35 mg/L brassinolide was added and the culture period was extended to 14 days, the capacity for meta-embryogenic expression increased by 52.98%. In genotype C, which showed a loss of embryogenicity, the addition of a high concentration (2.00 mg/L) of brassinolide and a 14-day culture period also led to a 32.79% increase in embryogenic expression. In a study conducted by Pullman et al. (2003), it was found that the addition of BL to the culture medium can increase the differentiation rate in calli of Loblolly Pine, Douglas-fir and Spruce calli. This addition also helps to differentiate between species that are normally difficult to distinguish, such as Loblolly Pine and Douglas-fir. Furthermore, the addition of BL to the process of somatic embryogenesis process was found to enhance its ability to undergo somatic embryogenesis (Mandava, 1988).

### 4.2 Effect of BL on the content of stored substances in embryogenic callus of Korean pine

Somatic cells are dependent on sugar as the primary energy source for cell metabolism and as a source of energy and carbon. Carbohydrates can also act as osmotic agents during somatic embryo development, helping to protect the integrity of the cell membrane and mitigate desiccation stress (Gomes et al., 2014). Studies have shown that the reduction in sugar content during somatic embryo development is related to the ability of somatic embryos to utilize sugars at different developmental stages, but there is no significant correlation with the number of somatic embryo transitions (Correia et al., 2012). In our study, no significant correlation was found between soluble sugars and the number of somatic embryo transitions. However, we observed that in the early stages of brassinolide addition to the genotype A embryogenic callus, the sugar content was significantly reduced, while the starch content increased. This suggests that the embryogenic callus of genotype A may experience a reduction in energy substances in the early stages of brassinolide addition. On the other hand, the sugar content in the embryogenic callus of genotype B increased while the starch content decreased. No significant change was observed in genotype C after the addition of brassinolide. Our results, therefore, suggest that the different genotypes have different coping strategies in response to the changes in energy partitioning after the addition of brassinolide.

The content of storage substances in conifers, especially carbohydrates, has a significant impact on somatic embryogenesis (Klimaszewska et al., 2004; Grigova et al., 2007). Carbohydrates are stored and accumulate in these trees, and the developing embryogenic tissues require a high level of energy metabolism during cell division and differentiation. Starch hydrolysis provides the necessary energy for this process (Martin et al., 2000; Pescador et al., 2008). Our studies have shown that the addition of brassinolide to callus with weak embryogenicity leads to an accumulation of starch, with more after 7 days than after 14 days. This result is consistent with previous studies. The callus of genotype B, which has strong embryogenicity, and the callus of genotype C, which has lost its embryogenicity, showed a relatively high consumption of starch. In genotype B, the accumulation of starch was significantly lower after the addition of a certain concentration of brassinolide. This indicates that brassinolide promotes cellular activities and increases the biological metabolism, which requires more energy. The high-level metabolism of the cells, therefore, requires more energy (Martin et al., 2000; Pescador et al., 2008).

The accumulation of soluble protein can serve as a source of material and energy for the development of embryogenic tissue, and thus promote its formation. In this study, we found that the protein content of three genotypes of embryogenic callus cultured with brassinolide showed no significant differences over different time periods. Similarly, no significant difference in protein content was observed during the differentiation and maturation stages of *Cordyline australis* somatic embryos of, supporting our results (Marzena Warchoł et al., 2015). Our analysis of soluble proteins in the embryogenic callus of Korean pine revealed a significant positive correlation with the number of somatic embryo transfers. This result is consistent with similar studies on *Gossypium hirsutum* (Liu and Yao, 1991) and *Fraxinus mandshurica* (Cong et al., 2012), which also showed a higher soluble protein content in the embryonic body during the row formation period. These results further support our view.

### 4.3 Effect of brassinolide on MDA content in embryogenic callus

MDA is formed after peroxidation of the cell membrane. The combination of MDA with the membrane enzymes leads to a series of physiological and biochemical dysfunctions and, in severe cases, can even lead to the death of the plant (Zhao et al., 1993). In stress situations, plants tend to produce a significant amount of MDA, which is often used as an indicator of the degree of cell damage. The experiment showed that the addition of brassinolide resulted in a higher MDA content in only a few concentrations of the three embryogenic calli than in the control group. In the other treatments, however, there was no significant difference in MDA content compared to the control group. This indicates that brassinolide did not cause significant damage to the plant cells. It was also observed that calli with strong embryogenicity had relatively low MDA content, while calli with loss of embryogenicity had relatively high MDA content. Studies suggest that a lower MDA concentration may help to slow callus senescence (Yang et al., 2013) and prevent loss of embryogenicity.

### 4.4 Effect of brassinolide on antioxidant enzyme activity in embryogenic callus

Plant cells have a comprehensive antioxidant system that regulates the level of reactive oxygen species (ROS) through antioxidant enzymes such as SOD, POD, and CAT. This system helps to maintain a dynamic balance of ROS in the cells and reduce their potential damage. SOD, the first enzyme in the antioxidant reaction, converts superoxide anions into hydrogen peroxide (Mittler, 2002). Increased SOD activity is beneficial for the development of early somatic embryos. The study showed that the addition of brassinolide to the callus with strong embryogenicity led to a significant increase in SOD activity in the early stage. In addition, the experimental group with a higher number of somatic embryo transitions also showed higher SOD activity, which supporting previous results. As the subculture cycle increased, SOD activity in the plants decreased significantly, which is consistent with the studies of Ren et al. (2022). In plants, the enzyme POD is responsible for the conversion of hydrogen peroxide into water and oxygen. A recent study showed that callus with strong embryogenic ability has higher POD activity. In addition, POD activity increased significantly as the callus grew and was cultured for longer periods of time, In a study with *Pinellia ternata*, it was observed that the addition of BL led to an increase in endogenous POD activity (Guo et al., 2022). The study also showed that there is a positive correlation between embryogenic ability and POD activity in the callus. Further studies have shown that the activity of the cell wall is closely related to POD activity, which in turn can influence the rate of cell growth and division (Marco and Roubelakis-Angelakis, 1996). POD activity of is closely related to respiration and energy metabolism, with higher levels indicating a greater capacity for embryogenesis. When POD activity decreases, metabolism slows down and the capacity for somatic embryogenesis gradually decreases. In our study POD activity in callus with high embryogenesis was found to be significantly higher than that in callus with low embryogenesis when proliferation time was prolonged. In addition, high CAT activity in embryogenic callus can attenuate the inhibitory effect of H_2_O_2_ on somatic embryogenesis (Liu et al., 2019). A study on *Crocus sativus* L. found that during the process of somatic embryo differentiation, the activity of antioxidant enzymes SOD and CAT increased in early development (Blazquez et al., 2009). In our study, it was found that callus with strong embryogenic ability showed an increase in CAT activity during development., In addition, the CAT activity of in callus of genotype A with weak embryogenic ability increased significantly after brassinolide treatment. However, CAT activity in the callus of genotype C with loss of embryogenicity did not show any significant changes.

### 4.5 Effects of brassinolide on IAA and ABA contents in embryogenic callus of Korean pine

Numerous studies have shown that auxin plays a crucial role in the conversion of somatic plant cells into embryogenic cells (Michalczuk et al., 1992; Yang et al., 2012; Xu et al., 2013). A sufficient amount of auxin is essential for the proper development and growth of somatic embryos. The concentration and ratio of endogenous hormones can regulate plant morphology and development. The ratio of IAA to ABA or other endogenous hormones can effectively indicate the ability of somatic embryogenesis of explants (Żur et al., 2015). It is well known that auxin hormones regulate callus proliferation and differentiation *in vitro* (Song et al., 2016). A study on *Abies nordmanniana* showed that callus with strong embryogenic ability had higher IAA levels than callus with weak embryogenic ability. In addition, the addition of auxin antagonists promoted the maturation of somatic embryos and increased the level of the endogenous hormone IAA (Find et al., 2002). Our study emphasized the importance of maintaining a high IAA content in embryogenic tissues and showed that in the control group without brassinolide, the IAA content was low in the callus with strong embryogenic. However, the addition of a low concentration of brassinolide was able to reduce the content of endogenous IAA in the callus, resulting in a higher yield of somatic embryos when the IAA content was low. This result is consistent with the previous point. In addition, a study also showed that after the addition of brassinolide, the yield of somatic embryos can be increased even when the IAA content is high (Suleman, 1994). The mechanism of BL-induced growth was found to be independent of auxin, but there is some synergistic effect between BL and auxin in promoting growth. Although exogenous brassinolide cannot increase the overall auxin content, it can regulate its content in various tissues (Martine et al., 1998).

In the study of *Schisandra incarnata* SE, ABA was found to play a crucial role in the maturation of cotyledon embryos. High levels of ABA were found to promote the maturation of somatic embryos (Sun et al., 2013). Our study found that a low concentration of brassinolide could increase the content of endogenous ABA in the callus. When the ABA content was high, the number of somatic embryo transitions was also higher, confirming the above observation. However, we found that in genotype A embryogenic callus, the addition of 0.75 mg/L brassinolide for culture resulted in a significantly lower ABA content than in the control group. Nevertheless, the number of somatic embryo transformations increased. Therefore, we speculate that some genotypes may attenuate the role of ABA in somatic embryo transformation after the addition of an appropriate concentration of BL. However, further studies are needed to confirm this hypothesis.

### 4.6 Effect of brassinolide on IAA:ABA ratio in embryogenic callus

In somatic embryogenesis of *Camellia sinensis* ‘Tieguanyin’, the content of endogenous IAA and ABA was found to be closely related to the development of spherical embryos and the maturation of somatic embryos (Guo et al., 2018). In addition, the ratio of IAA to ABA is a crucial factor influencing somatic embryogenesis in plants (Ren et al., 2022). The expression of callus embryogenicity benefits from a lower ratio of IAA: ABA (Liang et al., 2016). The study conducted on *Eucalyptus grandis* have shown that a lower ratio of IAA to ABA promotes somatic embryogenesis (Ouyang et al., 2014). In our study it was found that a higher number of somatic embryo transfers was often associated with a lower ratio of IAA: ABA, supporting the previous view. However, when the genotype A embryogenic callus was cultured with 0.75 mg/L brassinolide, the ratio of IAA: ABA increased, while the number of somatic embryos also increased. Moreover, after cultivation in brassinolide, the number of somatic embryos with a higher ratio of IAA: ABA was greater than those with a lower ratio. Further studies and analysis are needed to fully understand this phenomenon.

### 4.7 Effects of brassinolide on PAL activity and lignin content in embryogenic callus

PAL is the first enzyme in the phenylpropane metabolic pathway and is responsible for both the lignification of cells and the synthesis of plant cell walls (Wu et al., 2017). Research has shown that monitoring the expression of PAL in plant tissue can indicate the onset of callus differentiation (Ge et al., 2023). During the process of callus differentiation in the callus cultures of tobacco, stevia and salvia, PAL was discovered to play an important role in cell differentiation. This finding indicates a close correlation between PAL activity and the cellular differentiation process in callus cultures (Ouyang and Xue, 1988). In this study, PAL activity was found to be higher in the concentration gradient with a large number of somatic embryos after brassinolide treatment in genotype B, which has a strong embryogenic ability. PAL activity was also significantly higher in the concentration gradient with a large number of somatic embryo conversions, which is consistent with the above results. However, in genotype C, in which callus embryogenicity began to decline, the PAL activity was significantly higher in the experimental group than in the control group, but the yield of somatic embryos did not increase significantly. Therefore, it is suggested that PAL is involved in the physiological process of somatic embryo transformation during the transformation of Korean pine embryogenic callus. This finding may be helpful for the efficient transformation of somatic embryos into high-fit callus. However, increasing PAL activity in callus with low embryogenicity could not increase the number of somatic embryo transfers.

In this study it was found that in plant tissues with higher PAL activity, the secondary metabolite lignin is frequently formed during the metabolism of phenylpropane (Ouyang and Xue, 1988; Ge et al., 2023). The increase in lignin content was observed synchronously with the increase in PAL content, consistent with previous research results. Furthermore, the study showed that high levels of lignin content had a positive effect on somatic embryo transformation of genotype B embryogenic callus and promoted embryogenic transformation of genotype A under low-concentration brassinolide treatment. However, no obvious effect was observed for the gene of embryogenic loss of genotype C callus.

## 5 Conclusion

Our study showed that the addition of brassinolide during the culture stage of Korean pine embryogenic callus can increase its biological activity. The callus, with a more vital embryogenic ability, showed a higher physiological metabolism and consumed more soluble sugars and starch. The addition of brassinolide improved the physiological metabolism of the embryogenic callus and reduced the accumulation of storage substances. However, the addition of brassinolide had no significant effect on the soluble protein content. The study also found that the addition of brassinolide during proliferation culture could reduce the content of intracellular MDA, suggesting that it can reduce the toxic effect of ROS on embryogenic callus, improve the stress resistance of cells and increase the survival rate of embryogenic callus. It was also found that the activities of SOD, POD, and CAT increased after the addition of brassinolide at low concentrations, especially in embryogenic callus with strong embryogenic ability. This suggests that the addition of low concentrations of brassinolide may increase the adaptability of cells to unfavorable stress, which is beneficial for the survival and maintenance of embryogenic callus.

Brassinolide plays a crucial role in the hormonal regulation of callus and influences cell differentiation by regulating endogenous hormone levels. The addition of brassinolide increases PAL activity and lignin content, contributing to cell wall formation, cell division, and differentiation. In addition, low concentrations of brassinolide can reduce endogenous IAA content while increasing endogenous ABA content, thereby promoting somatic embryogenesis. However, the response of calli with different embryogenic capabilities varies at different concentrations of brassinolide. In some genotypes, higher concentrations of brassinolide can increase endogenous IAA content while decreasing ABA content, resulting in to improved embryogenicity. In addition, the addition of low concentrations of brassinolide to the embryogenic callus can increase intracellular PAL activity and lignin content and promote cell differentiation.

We conducted a series of propagation and culture programs to maintain Korean pine embryogenesis. First, we examined the embryogenic callus to determine the strengths and weaknesses of embryogenicity. Calli with low embryogenicity were subcultured after the addition of 0.75 mg/L brassinolide during proliferation culture. For callus with high embryogenicity, 0.35 mg/L brassinolide was added during the proliferation culture stage before subculturing. Callus with decreasing embryogenicity were subcultured after the addition of 2.00 mg/L brassinolide during the proliferation culture stage. This scheme effectively maintained the embryogenic ability of the callus while producing additional callus. In conifers, it is possible to perform a preliminary screening and determine the ability of the callus to form embryos. When evaluating the strength of somatic embryogenesis, we found that the addition of an appropriate concentration of brassinolide can enhance the expression of embryonic characteristics and increase the number of somatic embryos produced.

## Data availability statement

The raw data supporting the conclusions of this article will be made available by the authors, without undue reservation.

## Author contributions

SN: Writing – original draft, Data curation, Methodology, Writing – review & editing. YY: Data curation, Writing – original draft. YW: Data curation, Writing – original draft. SL: Writing – original draft, Formal analysis. WG: Writing – original draft, Formal analysis. LY: Methodology, Supervision, Writing – review & editing. HS: Conceptualization, Methodology, Supervision, Writing – review & editing.
